# Quantitative Analysis of the Recovery Process in Pure Iron Using X-ray Diffraction Line Profile Analysis

**DOI:** 10.3390/ma14040895

**Published:** 2021-02-13

**Authors:** Shota Sugiyama, Toshio Ogawa, Lei He, Zhilei Wang, Yoshitaka Adachi

**Affiliations:** Department of Materials Design Innovation Engineering, Graduate School of Engineering, Nagoya University, Furo-cho, Chikusa-ku, Nagoya 464-8603, Japan; sugisho@me.com (S.S.); he.lei@material.nagoya-u.ac.jp (L.H.); wang.zhilei@material.nagoya-u.ac.jp (Z.W.); adachi.yoshitaka@material.nagoya-u.ac.jp (Y.A.)

**Keywords:** recovery, dislocation substructure, pure iron, modeling, modified Williamson-Hall and Warren-Averbach methods

## Abstract

We conducted quantitative analysis of the recovery process during pure iron annealing using the modified Williamson-Hall and Warren-Averbach methods. We prepared four types of specimens with different dislocation substructures. By increasing the annealing temperature, we confirmed a decrease in dislocation density. In particular, screw-dislocation density substantially decreased in the early stage of the recovery process, while edge-dislocation density gradually decreased as annealing temperature increased. Moreover, changes in hardness during the recovery process mainly depended on edge-dislocation density. Increases in annealing temperature weakly affected the dislocation arrangement parameter and crystallite size. Recovery-process modeling demonstrated that the decrease in screw-dislocation density during the recovery process was mainly dominated by glide and/or cross-slip with dislocation core diffusion. In contrast, the decrease in edge-dislocation density during the recovery process was governed by a climbing motion with both dislocation core diffusion and lattice self-diffusion. From the above results, we succeeded in quantitatively distinguishing between edge- and screw-dislocation density during the recovery process, which are difficult to distinguish using transmission electron microscope and electron backscatter diffraction.

## 1. Introduction

Iron and steel are widely used in several applications owing to their low-cost and mass production. A key process in the design of iron and steel is mainly to control recovery and recrystallization in the annealing process. In particular, the interaction between recovery and recrystallization is important to control the microstructure during annealing.

The interaction between recovery and recrystallization during annealing in iron and steel was extensively investigated [1,2,3,4,5,6]. For low-carbon steel, Osawa et al. [1] reported that the formation of C–Mn dipoles leads to the retardation of recovery and changes in the recrystallization texture. Additionally, Belyakov et al. [2] demonstrated that the recrystallization progress slows down when recovery is rapid. This was because the recrystallization driving force decreased as a result of the accelerated recovery. In the case of pure iron, the relationship between three-dimensional recrystallized grains and the Avrami exponent for recrystallization was investigated [6]. The Avrami exponent was reported to correspond to the growth direction of the recrystallized grains [7]. In our previous study, the inconsistency between the theoretical and experiment results was confirmed, and this was probably because of the interaction between recovery and recrystallization. In particular, we pointed out that the recrystallization progress is retarded in the later stage of annealing because of the extremely rapid progress of recovery. Therefore, a precise understanding of the recovery process during annealing is crucial for analyzing recrystallization behavior.

Research into the recovery-process characteristics in pure iron showed that the softening caused by recovery is larger than that caused by recrystallization during annealing [8]. Furthermore, changes in dislocation substructure during the recovery process were investigated by transmission electron microscope (TEM) [9]. Recently, X-ray diffraction line profile analysis called the modified Williamson–Hall and Warren-Averbach methods was applied to quantitative analysis of dislocation substructures [10,11]. The modified Williamson-Hall and Warren-Averbach methods reveal information regarding dislocation substructures, such as dislocation density, dislocation character, the dislocation arrangement parameter, and crystallite size [12,13]. Quantitative analysis of the dislocation character, namely, edge- and screw-dislocation densities, is difficult to conduct using TEM and electron backscatter diffraction (EBSD). The mechanism of coalescence and annihilation of dislocations during the recovery process differs depending on the dislocation character. Therefore, the recovery process should distinguish between edge- and screw-dislocation densities. The modified Williamson-Hall and Warren-Averbach methods are suitable for conducting such analysis. The modified Williamson-Hall and Warren-Averbach methods were previously employed to investigate the recrystallization behavior of pure iron during annealing [14]. However, the previous report was a singular case because the pure iron was cold-rolled at a reduction rate of 99.8%. Moreover, the quantitative analysis of the recovery process, namely, the dislocation substructures, was insufficient. Thus, the recovery process during annealing in pure iron should be investigated in more detail using the modified Williamson-Hall and Warren-Averbach methods.

First, we investigated the recovery process of pure iron, neglecting the effect of alloying elements. Study findings should prove useful for various steel types. The purpose of the present study is to perform quantitative analysis of the recovery process during the annealing of pure iron using X-ray diffraction line profile analysis. Moreover, we sought to develop a new model of the recovery process in pure iron based on the quantitative analysis of dislocation substructures.

## 2. Materials and Methods

Just like in the previous study, we used pure iron here [6]. Vacuum-melted ingots were rough-rolled and hot-rolled in the austenite region to a thickness of 4.0 mm. The hot-rolled sheets were then cold-rolled to a thickness of 0.80 mm (a reduction of 80%). After cold rolling, specimens were heated to 623, 673, and 723 K at a rate of 10 K/s, held at that temperature for 10 s, and then water-quenched to room temperature (298 ± 2 K) within 2 s of being removed from the furnace. Recrystallization did not start in those specimens. During the experiments, the specimens were isothermally held at 823 K for various time periods to investigate the conditions for the beginning of recrystallization.

We conducted Vickers hardness tests (HMV-1, Shimadzu, Kyoto, Japan) under an applied load of 98 N for 10 s to evaluate the recovery process (ISO 6507). Standard deviation was calculated from the obtained results for the three specimens.

We evaluated the dislocation density, dislocation character (edge- and screw-dislocation densities), dislocation arrangement parameter, and crystallite size in each specimen by X-ray diffraction line profile analysis called the modified Williamson-Hall and Warren-Averbach methods. X-ray diffraction patterns of each specimen were obtained using an X-ray diffractometer (Ultima IV, Rigaku, Tokyo, Japan) with Cu Kα radiation of *λ* = 0.15418 nm wavelength at 40 kV and 40 mA (scanning speed: 0.1° min^−1^). Standard deviation was calculated from the obtained results for at least two specimens. Additionally, we conducted in situ measurements of electrical resistivity during annealing to evaluate the validity of the X-ray diffraction profile analysis because good correlation between electrical resistivity and dislocation density was observed [15]. We applied a current of 300 mA to the specimens and measured the voltage changes during annealing. Electrical resistivity was calculated using voltage values.

Microstructure observation and microtexture analysis at the quarter-thickness position in the rolling direction (RD)–normal direction (ND) plane were carried out with cold-rolled and annealed specimens using an electron backscatter diffraction/field emission scanning electron microscopy (EBSD/FEG-SEM) system (JSM-7001FA, JEOL, Tokyo, Japan) with OIM Analysis software (version 7.3.1, TSL solutions, Kanagawa, Japan). The step size of the EBSD measurements was 1 μm.

## 3. Modified Williamson-Hall and Warren-Averbach Methods

The modified Williamson–Hall method is expressed by the following equation [10], and the equation was constructed as functions of parameter *k* (=2sin*θ*/*λ*) and Δ*k* (=*β*cos*θ*/*λ*):

(1) $$\Delta {k\;=\;\alpha \;+\;\varphi kC}^{\frac{1}{2}}$$

where *α* is the parameter that depends on crystallite size, *φ* is a constant, and *C* is the average contrast factor. Additionally, diffraction angle *θ* and integral breadth *β* could be obtained in the X-ray diffraction peaks. Here, average contrast factor *C* is given by the following equation:

(2) $${C\;=\;C}_{h00}{(1\;–\;qH}^{2})$$

where *C_h00_* is the contrast factor in the crystal plane {*h00*}, *q* is the parameter that depends on dislocation character, and *H* is the orientation parameter. Substituting Equation (2) into Equation (1) gives Equation (3), and orientation parameter *H* is given by Equation (4) as a function of Miller index *{hkl}*:

(3) $${(\Delta k\;–\;\alpha )}^{2}{/k}^{2}{\;=\;\varphi }^{2}C_{h00}\left({1\;–\;qH}^{2}\right)$$

(4) $$H^{2}\;=\;\left(h^{2}k^{2}{+k}^{2}l^{2}{+l}^{2}h^{2}\right)/\;{\left(h^{2}{+k}^{2}{+l}^{2}\right)}^{2}$$

Equation (3) indicates a linear relationship between the left-hand side of Equation (3) and *H*^2^, and the optimal value of *α* is determined by optimizing linearity. Here, the slope of the (Δ*k* – *α*)^2^/*k*^2^ vs. *H*^2^ plots when the linearity is optimized corresponds to the value of *q*. Furthermore, the value of *α* is given by the following equation:

(5) $$\alpha \;=\;\frac{0.9}{D}$$

where *D* is the average crystallite size. Therefore, the average crystallite size can be estimated by determining the value of *α*.

The modified Warren–Averbach method is expressed by the following equation [10]:

(6) $${\ln A(L)\;=\;\ln A}^{S}(L)\;–\;\frac{{\pi b}^{2}{\rho L}^{2}}{2}\;\times \;\ln\left(\frac{R_{e}}{L}\right){\times \;(k}^{2}C)\;+\;Q\left(k^{4}C^{2}\right)$$

where *A*(*L*) is the real part of Fourier coefficient, *A^S^*(*L*) is the size of the Fourier coefficient, *L* is the Fourier length, *b* is the Burgers vector, *ρ* is the dislocation density, *R_e_* is the effective outer cut-off radius of dislocations, and *Q* is a constant. If the coefficient of the second term on the right side of Equation (6) is *Y*(*L*) = −(*πb*^2^*ρL*^2^/2) × ln(*R_e_*/*L*), the following equation is obtained:

(7) $${Y(L)/L}^{2}\;=\;–\;(\frac{{\pi b}^{2}\rho }{2}){\ln R}_{e}\;+\;(\frac{{\pi b}^{2}\rho }{2})\ln L$$

Equation (7) indicates a linear relationship between the left-hand side of Equation (7) and ln*L*. Thus, dislocation density *ρ* can be estimated from slope (=*πb*^2^*ρ*/2) of the *Y*/*L*^2^ vs. ln*L* plots when linearity is optimized. Moreover, edge- and screw-dislocation densities can be estimated using the values of *q* and *ρ*.

(8) $$\rho_{e}\;=\;\frac{2.64\;–\;q\;}{2.64\;–\;1.29}\;\times \;\rho$$

(9) $$\rho_{s}\;=\;\frac{q\;–\;1.29}{2.64\;–\;1.29}\;\times \;\rho$$

The values of *ρ_e_* and *ρ_s_* are the edge- and screw-dislocation densities, respectively. The value of *q* is 1.29 when all dislocations are edge dislocations, while the value of *q* is 2.64 when all dislocations are screw dislocations [16]. Thus, edge- and screw-dislocation densities can be estimated by Equations (8) and (9), respectively. Lastly, dislocation arrangement parameter M^*^ is given by the following equation:

(10) $$M^{*}{\;=\;R}_{e}\rho^{\frac{1}{2}}$$

## 4. Results and Discussion

### 4.1. Quantitative Analysis of Dislocation Substructure

**Figure 1:**
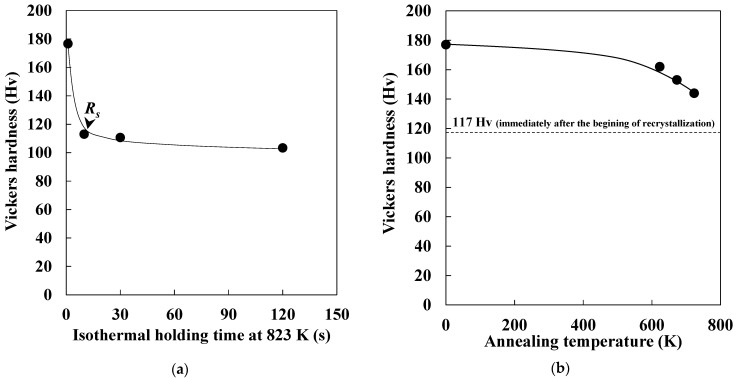
Changes in Vickers hardness as functions of (**a**) isothermal holding time at 823 K and (**b**) annealing temperature (*R_s_*: recrystallization starting time).

Figure 1a demonstrates the change in Vickers hardness during isothermal holding at 823 K. The drastic softening was confirmed during the early stage of isothermal holding, followed by gradual softening. The recrystallization started when the isothermal holding time at 823 K reached 10 s [6]. Therefore, the drastic softening was mainly attributed to the progress of recovery. The softening due to the recovery was reported to be larger than that due to the recrystallization during annealing in pure iron [8]. The obtained result in this study agrees with that previously reported. Figure 1b shows the change in Vickers hardness as a function of annealing temperature. Gradual softening was observed as annealing temperature increased. As shown in Figure 1a, Vickers hardness was approximately 117 Hv immediately after the beginning of the recrystallization. Accordingly, the gradual softening corresponded to the progress of recovery. This result indicated that four types of specimens at various stages of recovery were prepared.

**Figure 2:**
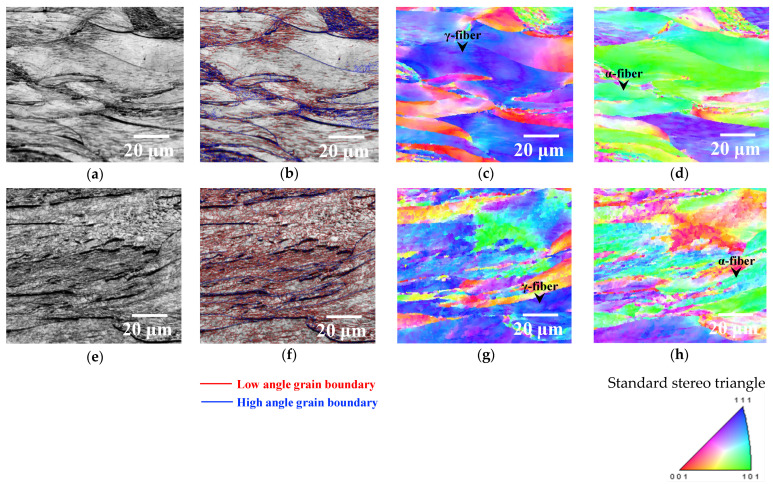
Image quality, grain-boundary misorientation angle, and normal direction (ND) and rolling direction (RD) orientation maps of specimens annealed at (**a**–**d**) 623 and (**e**–**h**) 673 K for 10 s.

**Figure 3:**
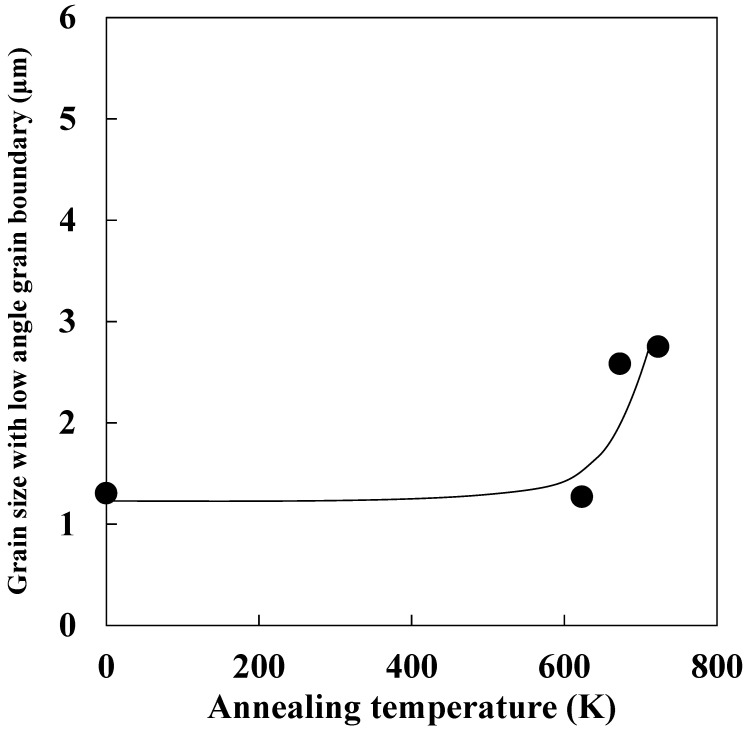
Changes in grain size with low-angle grain boundary during annealing.

Figure 2 shows the image quality map, grain boundary misorientation angle map, and ND and RD orientation maps for each specimen. For the specimen annealed at 623 K (Figure 2a–d), a major portion of microstructure consisted of non-re-crystallized grains (Figure 2a), and the fine grains surrounded by low-angle grain boundaries (red lines in Figure 2b) were partially observed within the non-re-crystallized grains. The fine grains within the non-re-crystallized grains may be attributed to the existence of dislocation cells and/or subgrains. It is possible that some small dislocation cells and/or subgrains were not detected because the step size of the EBSD measurements was 1 μm. Furthermore, as shown in Figure 2c,d, the textures of the non-re-crystallized grains were mainly γ-fiber (ND//{111}) and α-fiber (RD//<110>), and these are typical textures in cold-rolled iron and steel [3]. In contrast, the dislocation cells and/or subgrains were observed in the entire specimen annealed at 673 K regardless of texture (Figure 2e–h). This result suggests that recovery progressed as annealing temperature increased. Moreover, we previously reported that the formation of subgrains within non-re-crystallized grains with both γ and α fiber occurred before the beginning of recrystallization [6], and the obtained result in the previous study is consistent with that reported in this study. The change in average grain size with a low-angle grain boundary during annealing is shown in Figure 3. The average size of grains with low-angle grain boundaries increased with annealing temperature. Therefore, recovery progressed as annealing temperature increased.

**Figure 4:**
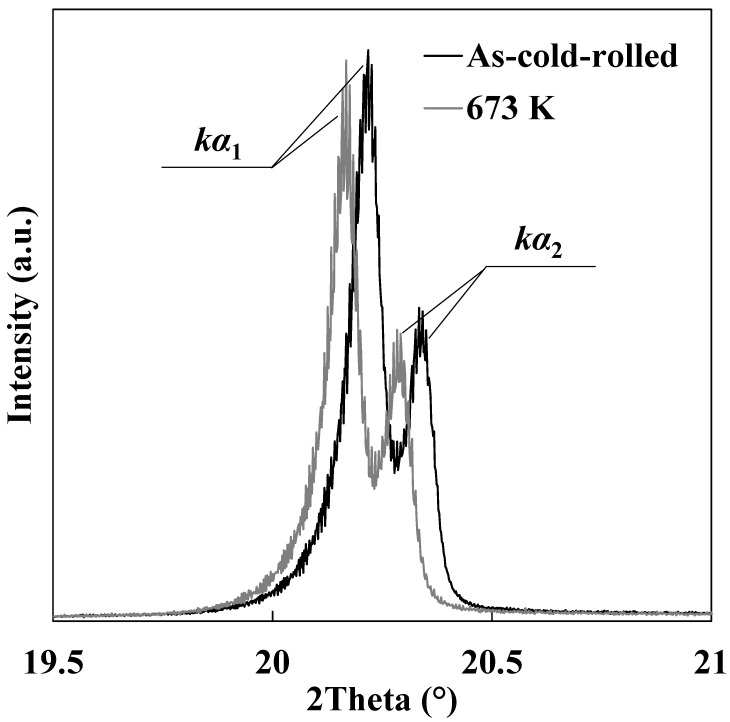
Typical X-ray diffraction patterns of cold-rolled and annealed specimens (crystal plane: {110}).

**Figure 5:**
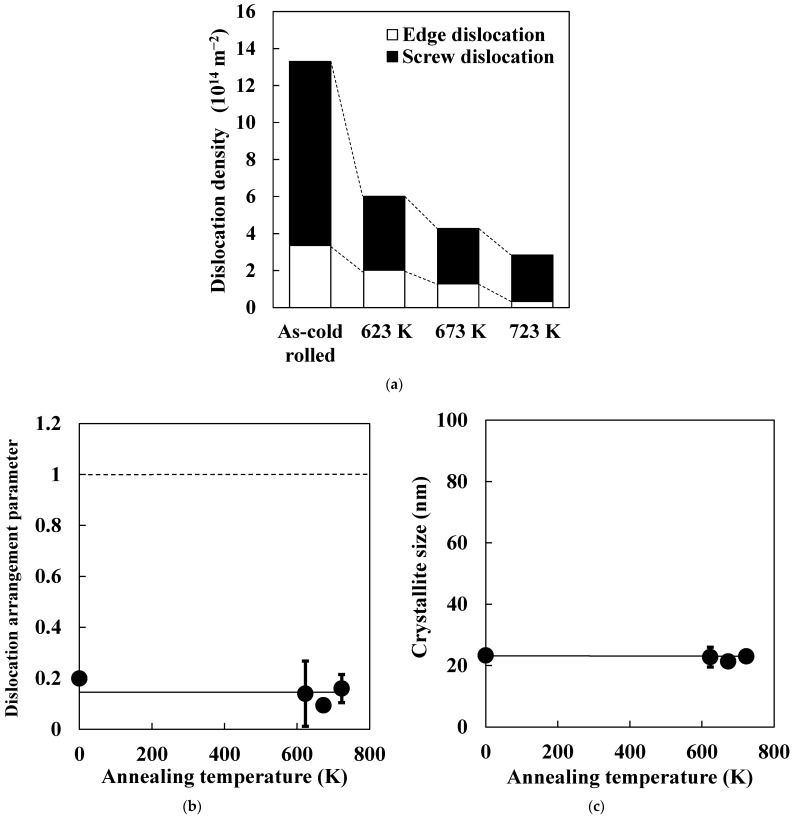
Changes in (**a**) edge- and screw-dislocation densities, (**b**) dislocation arrangement parameter, and (**c**) crystallite size in specimens as function of annealing temperature.

Figure 4 shows typical X-ray diffraction patterns of the cold-rolled and annealed specimens (crystal plane: {110}). There were two peaks (*kα*_1_ and *kα*_2_), and only the *kα*_1_ component was used for analysis after the elimination of the *kα*_2_ component using a Voigt function. The peak of the cold-rolled specimen appeared at a higher angle compared to that of the annealed specimen. Additionally, integral breadth was decreased by annealing. Figure 5 shows changes in edge- and screw-dislocation densities, dislocation arrangement parameter, and crystallite size during annealing in each specimen. Total dislocation density decreased as annealing temperature increased (Figure 5a). In the case of pure iron, Tomita et al. [14] reported that total dislocation density was approximately 2.0 × 10^14^ m^−2^ immediately before the beginning of recrystallization. Total dislocation densities in this study were larger than 2.0 × 10^14^ m^−2^ regardless of annealing temperature. Thus, the result shown in Figure 5a matches that obtained in the previous study. Additionally, the decrease in total dislocation density was significant during heating from room temperature to 623 K. As shown in Figure 5a, screw-dislocation density preferentially decreased in the early stage of the recovery process, and edge-dislocation density gradually decreased as annealing temperature increased. Furthermore, the screw-dislocation ratio hardly changed in the later stage of the recovery process. Screw dislocation preferentially coalesces and annihilates by glide and/or cross-slip, and edge dislocation then coalesces and annihilates by a climbing motion during later recovery stages. Thus, the decrease in total dislocation density was pronounced during heating from room temperature to 623 K, and it was mainly attributed to the coalescence and annihilation of screw dislocation. Dislocation density was measured by TEM in previous studies [17,18,19], but it was difficult to estimate edge- and screw-dislocation densities. Moreover, changes in edge- and screw-dislocation densities during deformation were previously reported [20], whereas changes during recovery were barely demonstrated. The present study quantifies changes in the ratio of edge and screw dislocations during the recovery process.

Dislocation arrangement parameter and crystallite size hardly changed as annealing temperature increased (Figure 5b,c). Studies reported that dislocation distribution is random when the value of the dislocation arrangement parameter is larger than 1 [21]. In contrast, dislocation interactions were large for dislocation-arrangement-parameter values smaller than 1. Thus, the result shown in Figure 5b indicates that dislocation interactions were large at every recovery stage. Moreover, we observed no correlation between cold reduction rate and crystallite size [22]. This finding implies that a change in dislocation substructures does not necessarily correlate with crystallite size, as the latter is weakly affected by increases in cold reduction rate. As shown in Figure 3 and Figure 5c, subgrain size increased as annealing temperature increased, whereas crystallite size hardly changed. This means that changes in subgrain sizes do not necessarily correlate with crystallite size because the latter is weakly affected by increases in annealing temperature. Therefore, it is difficult to estimate the degree of the recovery progress from the values of dislocation arrangement parameter and crystallite size.

**Figure 6:**
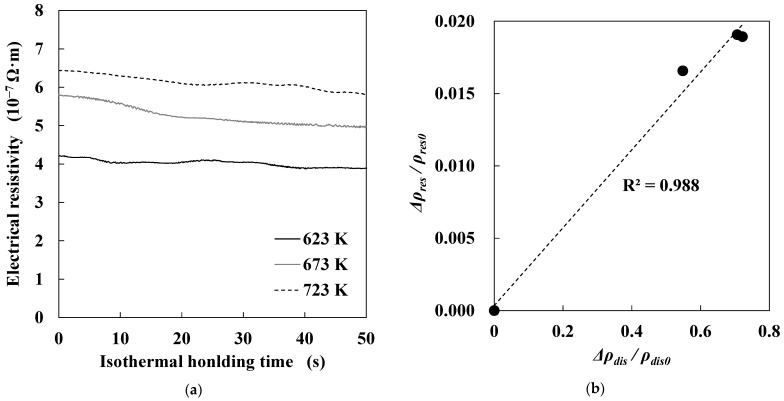
(**a**) Changes in electrical resistivity during isothermal holding at each temperature; (**b**) relationship between decreasing rates of electrical resistivity and dislocation density due to annealing.

Figure 6a shows changes in electrical resistivity during isothermal holding at each temperature level. Electrical resistivity gradually decreased with isothermal holding time regardless of annealing temperature. The correlation between electrical resistivity and dislocation density is well-known [15]. Thus, the decrease in electrical resistivity was attributed to the decrease in dislocation density due to the progress of recovery. Figure 6b demonstrates the relationship between decreasing rates of electrical resistivity and dislocation density due to the progress of recovery. *ρ_res0_* is electrical resistivity when specimens were heated to each target temperature, and Δ*ρres* is the decrease in electrical resistivity during isothermal holding for 10 s at each target temperature. Thus, Δ*ρ_res_/ρ_res0_* is the decreasing rate of electrical resistivity during isothermal holding for 10 s at each target temperature. Moreover, *ρ_dis0_* is dislocation density of as-cold rolled specimens, and Δ*ρ_dis_* is the decrease in dislocation density during annealing at each target temperature. Therefore, Δ*ρ_dis_/ρ_dis0_* is the decreasing rate of dislocation density during annealing at each target temperature. The decreasing rates of electrical resistivity and dislocation density showed good correlation, where the coefficient of determination (*R*^2^) was approximately 0.988. This result means that in situ electrical-resistivity measurements during annealing validated the total dislocation-density values shown in Figure 5a.

Ultimately, we discuss the relationship between hardness and dislocation substructure. As mentioned above, screw-dislocation density preferentially decreased in the early stage of the recovery process, and edge-dislocation density gradually decreased as annealing temperature increased. Moreover, we observed gradual softening as annealing temperature increased. These results suggest correlation between hardness and edge-dislocation density. The ratio of edge dislocation increases with thickness reduction by cold rolling in ferrite single-phase steel [20]. The relationship between dislocation strengthening and dislocation character is not necessarily clear, but the previous report implied that dislocation strengthening mainly depends on edge-dislocation density. Thus, hardness changes during the recovery process may depend on changes in edge-dislocation density. However, the effect of the distribution of dislocations on hardness was not considered in this study, and it should be further investigated in the future.

### 4.2. Recovery-Process Modeling

The recovery process is expressed by the following equation [23]:

(11) $$\frac{d\sigma }{dt}\;=\;-\frac{64}{{9M}^{3}\alpha^{2}}\frac{\sigma^{2}}{E}{\;v}_{D}\;\exp\left(-\frac{Q}{k_{B}T}\right)\sin h\left(\frac{\sigma V}{k_{B}T}\right)$$

where *σ* is stress due to dislocations, *E* is Young’s modulus, *M* is the Taylor factor, *v_D_* is the Debye frequency, *α* is a constant, *Q* is the activation energy for recovery, *T* is temperature, *V* is the activation volume, and *k_B_* is the Boltzmann constant. Additionally, dislocation density can be estimated by the following equation [23]:

(12) $$\rho \;=\;(\frac{\sigma }{M\alpha Gb})2$$

where *ρ* is dislocation density, *G* is the shear modulus, and *b* is the Burgers vector. The temperature dependency of the shear modulus for ferrite is given by the following equation [24]:

*G* = 64,000 (1 − 0.00044 (*T* − 300)) − 0.032 (*T* − 573)^2^ (13)

**Figure 7:**
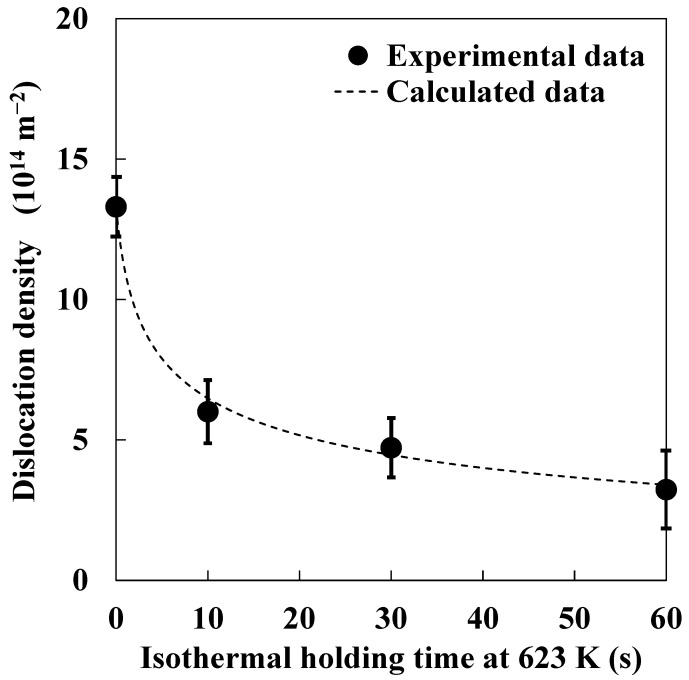
Changes in total dislocation density during isothermal holding at 623 K estimated by X-ray diffraction line profile analysis and the new model.

For modeling the experiment results, we used *Q* and *V* as fitting parameters. The values of other factors used in Equations (11) and (12) are summarized in Table 1. To estimate the values of *Q* and *V*, some specimens were isothermally held at 623 K for various periods; then, total dislocation density, and edge- and screw-dislocation densities in each specimen were estimated by X-ray diffraction line profile analysis. As a result, the estimated values of *Q* and *V* at 623 K were approximately 170 kJ·mol^−1^ and 1.75 × 10^28^ m^3^, respectively. Figure 7 shows changes in total dislocation density during isothermal holding at 623 K, estimated by both X-ray diffraction line profile analysis and the newly developed model. Total dislocation densities estimated by X-ray diffraction line profile analysis and the model showed a good correlation.

**Figure 8:**
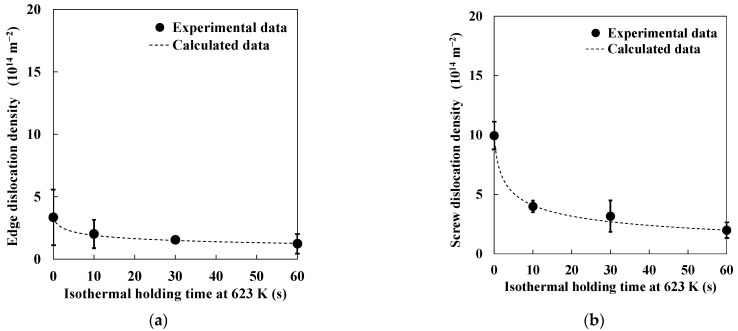
Changes in (**a**) edge- and (**b**) screw-dislocation densities during isothermal holding at 623 K, estimated by X-ray diffraction line profile analysis and the newly developed model.

As mentioned above, changes in edge- and screw-dislocation densities during the recovery process were quantified. Therefore, the values of *Q* and *V* could be estimated when *ρ* in Equation (2) was assumed to be the edge- or screw-dislocation density. Table 2 shows the estimated values of *Q* and *V* at 623 K when *ρ* in Equation (2) was assumed to be the edge- or screw-dislocation density. Furthermore, Figure 8 shows changes in edge- and screw-dislocation densities during isothermal holding at 623 K estimated by both X-ray diffraction line profile analysis and the newly developed model. Edge- and screw-dislocation densities estimated by X-ray diffraction line profile analysis and the model also showed good correlation. In the case of screw dislocation, the value of *Q* was close to that for dislocation core diffusion (174 kJ·mol^−1^ [26]), which means that the decrease in screw-dislocation density during the recovery process was mainly dominated by glide and/or cross-slip with dislocation core diffusion. The activation energy in the early stage of the recovery process was previously reported to be approximately 170 kJ·mol^−1^ [23,27]. These values are consistent with those obtained in this study. Furthermore, the value of *Q* when *ρ* in Equation (12) was assumed to be edge-dislocation density was larger than that when *ρ* in Equation (12) was assumed to be screw-dislocation density. Activation energy for lattice self-diffusion was reported to be 251 kJ·mol^−1^ [26]. The value of *Q* when *ρ* in Equation (12) was assumed to be edge-dislocation density was between that for dislocation core diffusion and lattice self-diffusion, which means that the decrease in edge-dislocation density during the recovery process was dominated by climbing motion with both dislocation core diffusion and lattice self-diffusion. Humphreys et al. [28] indicated that the climbing motion of edge dislocation was expected to be controlled by self-diffusion during the recovery process at high temperature levels. Smith et al. [23] also reported that activation energy for recovery in C–Mn steel gradually increased as annealing temperature increased. Thus, it is possible that the decrease in edge-dislocation density during the recovery process at higher temperature levels was governed by a climbing motion with lattice self-diffusion. The effect of annealing temperature on activation energy should be further investigated.

Several studies reported the relationship between dislocation density and thermal diffusivity [23,27,29]. However, these studies did not distinguish between edge- and screw-dislocation densities. Owing to the synergistic effect of the quantitative analysis and modeling of the recovery process, our study succeeded in both quantifying edge- and screw-dislocation densities, and in determining the activation energy and dominant factor for recovery.

## 5. Conclusions

We conducted quantitative analysis of the recovery process of pure iron during annealing using the modified Williamson-Hall and Warren-Averbach methods, and obtained the following results.

1. Dislocation-density decrease was confirmed by increasing annealing temperature. In particular, screw-dislocation density remarkably decreased during heating from room temperature to 623 K, while edge-dislocation density gradually decreased as annealing temperature increased.
2. Dislocation arrangement parameter and crystallite size hardly changed as annealing temperature increased.
3. Changes in hardness during the recovery process mainly depended on edge-dislocation density.
4. A new model for the recovery process based on X-ray diffraction line profile analysis was developed. Using the new model, we demonstrated that the decrease in screw-dislocation density during the recovery process was mainly dominated by glide and/or cross-slip with dislocation core diffusion. In contrast, the decrease in edge-dislocation density during the recovery process was dominated by a climbing motion with both dislocation core diffusion and lattice self-diffusion.

## Figures and Tables

**Table 1:** Values of factors used in Equations (11) and (12) [23,25].

*M*	*α*	*E* (Pa)	*v_D_* (s^−1^)	*k_B_* (J·K^−1^)	*b* (m)
2	0.3	2.05 × 10^11^	9.79 × 10^12^	1.38 × 10^−23^	2.5 × 10^−10^

**Table 2:** Estimated values of activation energy and activation volume at 623 K when *ρ* in Equation (12) is assumed to be edge- or screw-dislocation density.

*ρ*	Edge Dislocation Density	Screw Dislocation Density
Activation energy (kJ·mol^−1^)	186	167
Activation volume (m^3^)	5.60 × 10^28^	1.98 × 10^28^

## Data Availability

The data presented in this study are available on request from the corresponding author.
